# The role of Magnetic Resonance Images (MRIs) in coping for patients with brain tumours and their parents: a qualitative study

**DOI:** 10.1186/s12885-021-08673-z

**Published:** 2021-09-10

**Authors:** Natalie Tyldesley-Marshall, Sheila Greenfield, Susan J. Neilson, Martin English, Jenny Adamski, Andrew Peet

**Affiliations:** 1 Institute of Cancer and Genomics, University of Birmingham, Birmingham, B15 2TT UK; 2 Institute of Applied Health Research, University of Birmingham, Birmingham, B15 2TT UK; 3 Birmingham Children’s Hospital, Steelhouse Lane, Birmingham, B4 6NH UK; 4 School of Nursing, Institute of Clinical Sciences, University of Birmingham, Birmingham, B15 2TT UK

**Keywords:** Qualitative research, Patient views, Imaging, Oncology, Coping strategies, Patient families, Paediatric, CYP, Tumours

## Abstract

**Background:**

When children and young people (CYP) are diagnosed with a brain tumour, Magnetic Resonance Imaging (MRI) is key to the clinical management of this condition. This can produce hundreds, and often thousands, of Magnetic Resonance Images (MRIs).

**Methods:**

Semi-structured interviews were undertaken with 14 families (15 parents and 8 patients), and analysed using Grounded Theory. Analysis was supported by the Framework Method.

**Results:**

Although the focus of the research was whether paediatric patients and their families find viewing MRIs beneficial, all patients and parents discussed difficult times during the illness and using various strategies to cope. This article explores the identified coping strategies that involved MRIs, and the role that MRIs can play in coping. Coping strategies were classified under the aim of the strategy when used: ‘Normalising’; ‘Maintaining hope and a sense of the future’; ‘Dealing with an uncertain future’; and ‘Seeking Support’.

**Conclusions:**

Coping and finding ways to cope are clearly used by patients and their families and are something that they wish to discuss, as they were raised in conversations that were not necessarily about coping. This suggests clinicians should always allow time and space (in appointments, consultations, or impromptu conversations on the ward) for patient families to discuss ways of coping.

MRIs were found to be used in various ways: to maintain or adapt normal; maintain hope and a sense of the future; deal with an uncertain future; and seek support from others. Clinicians should recognise the potential for MRIs to aid coping and if appropriate, suggest that families take copies of scans (MRIs) home. Professional coaches or counsellors may also find MRIs beneficial as a way to remind families that the child is in a more stable or ‘better’ place than they have been previously.

**Supplementary Information:**

The online version contains supplementary material available at 10.1186/s12885-021-08673-z.

## Background

Brain tumours are the most common solid tumours in children, and the leading cause of childhood cancer-related deaths [1]. Approximately 400 children are diagnosed each year in the United Kingdom [2]. Magnetic Resonance Imaging (MRI) is essential to the clinical management of children and young people (CYP) with this chronic illness [3].

In Bury’s (1991) seminal article, Bury described how those with chronic illnesses negotiate reality subsequent to their diagnosis to “manage, mitigate, or adapt to” the impact on their lives; in other words, to “cope”; and defined “what people do” in order to cope, as “coping strategies” [4] (pp.452–461). Recent conceptualizations of coping [5–7] and definitions of coping strategies vary widely [5], so this article takes a broader definition of “coping strategies” as any “cognitive or behavioural techniques to cope with the physical and psychological challenges” [8] (p.36) being experienced. Past studies have found that seeing the images produced by MRI - Magnetic Resonance Images (MRIs) - can provide reassurance [9–11], and hope for patients [9, 12] and their parents [9], although there is scant research on the role MRIs might play in coping. This article aims to add to existing knowledge by detailing specific coping strategies of CYP with brain tumours and their parents which involve MRIs.

## Methods

### Research design

The research design was cross-sectional, as the research was concerned with similarity and difference between individual participants and families [13]. Qualitative methods were chosen as they are ideal to use to understand the world from the participant’s point of view, and when little is known about a topic [14]. Semi-structured interviews were chosen, allowing answers to be compared, yet flexibility to explore unexpected answers [15].

### Participants

Paediatric patients with brain tumours, and their parents, were recruited from a UK children’s hospital. For the family to be eligible, they had to have a child with a brain tumour diagnosed at least 3 months prior to being approached; and be deemed by their clinician not to be going through an acutely challenging period. The patients had to be either: on active treatment (or within 3 months of the end of treatment); or undergoing MRI surveillance of residual tumour. Patients with low-grade tumours treated with complete surgical excision were therefore not included. Participants needed to be able to see a visual prompt, a printout of another patient’s brain tumour MRI image (anonymised), and respond verbally to questions. Patients with severe learning difficulties, and those under 8 years, were excluded due to anticipated communication difficulties, although their parents were eligible.

Maximum variation sampling was used to provide the “broadest practicable range of participants” [16] (p.564) from the relatively limited number of potential participants. (Further details and the results of this research can be found elsewhere [9]).

### Data collection

All subjects gave their informed consent for inclusion before they participated in the study. The protocol was approved by the West Midlands - Black Country Research Ethics Committee (WM/16/WM/0490).

Child participants were met on two occasions, a ‘getting to know’ session, and then a semi-structured interview. It was hoped that this would be less daunting for young participants; as well as allowing researcher and patient to be more familiar and comfortable with each other, and so enhance rapport, resulting in richer and more detailed participant responses [17, 18]. On both occasions, the child participant’s parent was present.

Interviews were conducted from May 2017 to March 2018, using an interview schedule (see Appendix A). Questions were informed by clinical experiences of the paediatric oncologists caring for the patients, literature review, and the Research Advisory Group (parents of current and former patients), then piloted with the target population. Topics covered the emotional impact from first seeing MRIs; what was understood from the MRIs; and advantages and disadvantages from being able to see these.

A total of 14 families were interviewed: 15 parents (13 mothers) and 8 patients. Patients’ mean age was 12 years (range 8 to 15) and participant characteristics can be found in Table 1. All interviews were conducted by the first author (a female Research Fellow, with a Masters in Social Science, and many years’ experience in sociological research). Interviews were held at the venue of the parent’s choice (nine at their home, and five at the hospital) and on average lasted approximately 38 min (range 8 to 80).

**Table 1:** Participant characteristics

Patients	(***n*** = 8)	Parents (***n*** = 15) Families (***n*** = 14)	
**Characteristics**	***n***	**Characteristics**	***n***
*Gender*		*Gender*	
Female	4	Female	13
Male	4	Male	2
*Percentage of deprivation (Assessed by the Index of Multiple Deprivation (IMD))*			
10% least deprived	1	10% least deprived	2
20% least deprived	2	20% least deprived	2
30% least deprived	0	30% least deprived	0
40% least deprived	0	40% least deprived	1
50% least deprived	2	50% least deprived	2
50% most deprived	1	50% most deprived	3
40% most deprived	0	40% most deprived	0
30% most deprived	1	30% most deprived	2
20% most deprived	0	20% most deprived	0
10% most deprived	1	10% most deprived	3
*Age (years)*		*Age of child (years)*	
Under 8	(Interviewed parent only)	Under 8	6
8–12	4	8–12	4
13–15	4	13–15	4
*Ethnicity*		*Ethnicity of child*	
Caucasian	7	Caucasian	10
Asian	1	Asian	2
Mixed	0	Mixed	1
Unknown	0	Unknown	1
**Diagnosis**		**Diagnosis of child**	
Unbiopsied low grade glioma in child with Neurofibromatosis Type 1 (NF1)	2	Unbiopsied low grade glioma in child with Neurofibromatosis Type 1 (NF1)	4
Pilocytic astrocytoma	3	Pilocytic astrocytoma	3
Medulloblastoma	2	Medulloblastoma	3
Unbiopsied low grade glioma in child with no NF1	1	Unbiopsied low grade glioma in child with no NF1	2
		Langerhans cell histiocytosis of the brain	1
		Subependymal giant cell astrocytoma	1
*Years since diagnosis*		*Years since child’s diagnosis*	
< 1 year	1	< 1 year	3
1 < 2 years	1	1 < 2 years	2
2 < 5 years	0	2 < 5 years	2
5 < 10 years	4	5 < 10 years	5
> 10 years	2	> 10 years	2
*Age at diagnosis*		*Child’s age at diagnosis*	
< 1 year	0	< 1 year	2
1 < 2 years	2	1 < 2 years	3
2 < 5 years	2	2 < 5 years	4
5 < 10 years	3	5 < 10 years	4
> 10 years	1	> 10 years	1

### Data analysis

The transcripts were analysed using a Grounded Theory methodology [19], supported by the Framework Method [20]. Data analysis began with the first interview and continued throughout data collection.

The transcripts were primarily coded by NT, who first familiarised themselves with the data by transcribing each interview verbatim, and integrating contextual or reflective notes [20] (field notes) taken immediately after the interview into the transcripts. In one case, the parent declined the audiorecorder so only handwritten notes were taken, typed up straightaway, and combined with field notes.

At the beginning of analysis, early transcripts (anonymised and with contextual identifiers removed) were reviewed by other authors with different disciplinary backgrounds: AP (male professor in Paediatric Oncology / clinician), SG (female professor in Medical Sociology), and SN (female lecturer in Nursing / former palliative nurse); a male clinician; and the Research Advisory Group (RAG) (parents of current and former patients) [20].

NT coded transcripts line-by-line, using gerunds in coding in order to focus on processes, stay close to the data, and “ground themself” in each participant’s viewpoint [19]. Data from each code were compared for each interview, and between interviews (constant comparative method) to develop focused codes. Data management and analysis was facilitated by NVivo. Following the Framework Method, an additional step was added to the analysis. Extracts from each transcript were added into a framework matrix, a spreadsheet with each participant mapped against each code, to allow for more systematic comparison of codes [20]. Codes, categories and concepts were discussed and developed throughout the process; no preconceived codes were applied to the data [19]. It was concluded that categories were saturated when it appeared that further interviews would “no longer spar [k] new theoretical insights, nor revea [l] new properties of” the categories [19] (p.113).

Coping was not an intended focus of the research, however, early in the analysis it was realised that coping with the difficult times during the illness was discussed by all participants - parents and patients. NT was exploring literature on coping strategies throughout data collection and analysis, in order to enhance her understanding of concepts, and her sensitivity to these in the interviews and transcripts [19]. Theoretical sorting of the analytical memos and diagramming were undertaken to refine comparisons between categories, and illuminate relationships between them [19]. While touched on in a past publication [9], ‘coping and coping strategies’ was not covered in the article in order to fully expand upon it separately.

## Results

**Fig. 1:**
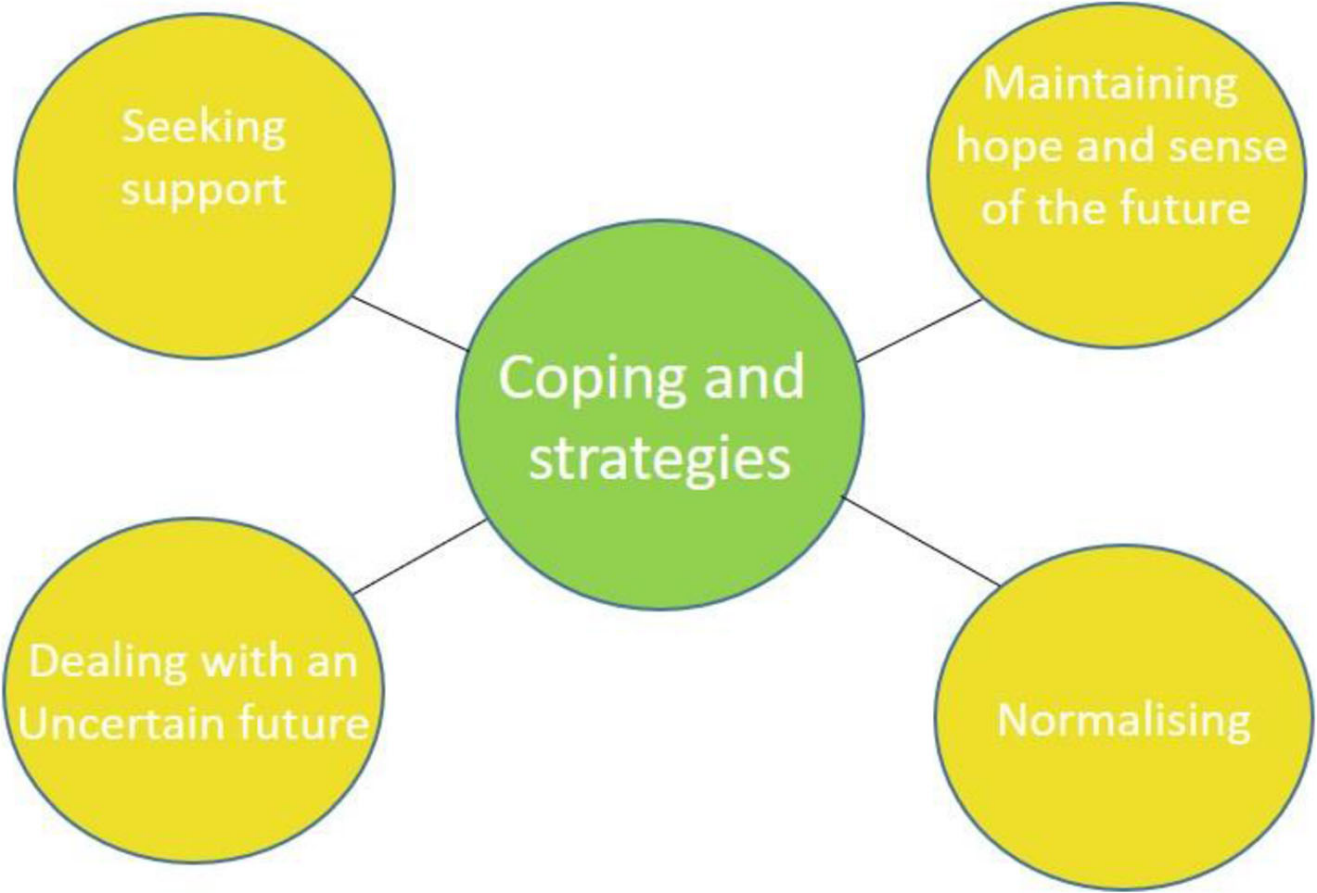
‘Coping and strategies’, and related categories

Coping and strategies were sometimes discussed in response to being asked how the participants found the wait to receive the results, though more often in response to questions about the value of MRIs. Typically, they were mentioned throughout the interview, but not recognised or referred to as coping strategies. Coping strategies were classified towards their ultimate aim: helping the patient maintain their self-worth and some sense of normality (‘Normalising’); maintaining hope for the future; or creating a future that they could view positively (‘Maintaining hope and a sense of the future’); reducing their anxiety or fear facing a future that can be highly unpredictable (‘Dealing with an uncertain future’); or ‘Seeking support’ (Fig. 1).

Quotations from participants are followed by a ‘P*x*’ which denotes a randomly assigned number for the participant interview. ‘[ …]’ highlights where the extract has been edited for clarity or brevity. Speakers are Child (C), Parent (P) or the interviewer (NT).

### Normalising

#### Maintaining normal

Bury (1991) describes normalisation as a way of coping. One form of normalisation is a “psychological ‘bracketing off’ of the impact of the illness” [1] (p.460); minimising the changes and disruption from the chronic illness, the treatment, and their effects, to give the impression that one’s ‘normal life’ has not changed, or has not changed significantly. By not talking about, or thinking about the tumour, or treatment, in spaces outside of the hospital, some participants were aiming to maintain a boundary, and not “*bring it home*” with them. When posed the question about whether they would like a copy of the image from their MRI to take home after a hospital appointment, many young patients did not want to do so, in order to “*not think about it*” until their next appointment. Two families mentioned keeping their child’s illness a secret from everyone except close family and friends, as they did want to change others’ perceptions of them, or be recipients of ‘pity’.

“*[I wouldn’t want an MRI image home at the end of the consultation] Just, ‘cosss’ … it’s, not somefink I’d probably look at. So I’d probably just, take it home, an’, sort of, not leave, well not just forget about it, but in a way, yer, I would. [ … ] it’s just -. ‘cos’ as long as I know it’s alright, I’ll just try to just continue with everything else, rather than bring it home. I just try to continue*.” P2, 13 – 15 year old patient

“*I don’t need to keep looking at it [the MRI image]. [ … ] we kinda forgot about it as best we can until the next appointment*.” P10, Parent of patient aged under 8

In a similar vein, two patients symbolically separated the tumour from themselves, by naming and therefore identifying it as a distinct entity. One referred to their tumour as their “*seed*”; while another (somewhat confusingly) named the remnants of their tumour “*Brain*” and “*Tumour*”.

“*C: And I think why I do, why I’m going to hospital, is for my ‘seed’.*” P5, 8 – 12 year old patient

#### Adapting normal

Some patients and parents adapted their sense of what was normal. They described the tumour, MRIs, appointments, scan results, and treatment/s as “*just normal*”, or normal for them, as this was all they could remember, or had been so much a part of their child’s life.

“*P: You get used to it [the tumour] over the years. It’s um, it’s quite normal really. It’s just a normal thing in your head now, isn’t it? To us. An everyday thing. [C: Yeah.] So it’s not frightening for us*.” P7, 13 – 15 year old patient

“*I feel better seeing the scan than just being told about the scans, so I think as time comes on, it will become a bit more of a norm. I know it sounds a bit of an awful thing to say [NT: Mmmm. NT laughs] that a brain scan is gonna be a norm, but yaknow at least we know what to expect when we go to the appointments*.” P10, Parent of patient aged under 8

### Maintaining hope and a sense of the future

#### Maintaining hope

For some participants, the MRIs served as a source of hope about the future. Participants referred to how being able to look back at earlier MRIs when the tumour was much larger before a treatment, or when it appeared to be spreading faster, and comparing that point to the present gave them hope, helping them get through the hard times.

“*There’s been times in the last couple of years where [Child]’s found it really hard, the tumour’s growing, it’s really bad. And [they’re] really stressed. But because we’ve got... images from [their] diagnosis, and although it is bad when it’s growing, you look back, and it’s nowhere near, nowhere near, that … huge mass that [they] had then*.” P12, Parent of 13 – 15 year old patient

“*C: Yeah. [I’d want to have a copy of my MRI [image] at the end of each consultation] It’d be like … , ‘Look how far I’ve come’.*” P8, 8 – 12 year old patient

#### Focusing on the positive

Some of the participants reported that although experiencing treatment or symptoms was difficult at times, it was worthwhile in the longer-term due to, for example, improving the prognosis, life expectancy, or quality of life. Some obtained copies of MRIs shown to them or said they would like to take a copy of the MRIs home, to serve as a visual reminder of this improvement.

“*And ummm, you easily forget the scans that you’ve seeeen, in the beginning, and then how much [the treatment]‘s helped, and the size of it exactly, and just knowing that all that painnn, tearsss, vomiting, [NT laughs] and sleepless nights and everything, it was just, kind of worth it...*” P4, Parent of patient aged under 8

“*P: Yeah. [Wants to take copies home of all of the MRIs] [C: All of them.]] ‘Cos’ it’s hard to go through treatment, especially … the first 18 months of [Child’s] [ … ] And I think … at times when you’re having a bad day, maybe [they] could have looked at that scan and compared and thought ‘Look. It actually is doing something’.*” P5, 8 – 12 year old patient

### Dealing with an uncertain future

#### Becoming ‘experts’

Due to their repeated exposure to medical terms and sometimes a number of different treatments, patients often became ‘experts’ in their illness and treatment/s [21], as did their parents. All but one parent described in great detail their child’s medical history, variation in their child’s symptoms, and / or treatment. Many parents had a strong understanding of (or at least familiarity with) hospital processes, procedures and lexicon, especially around viewing images; what one of our RAG termed a “*seasoned traveller*”. Some patients also demonstrated familiarity with their medical history, their images and / or medical lexicon.

“*I am shown, the image. I, I think I’ve, I’ve gathered a little bit of um common sense for it [viewing MRIs], even though I couldn’t, [Snorts] yaknow understand exactly what it is.*” P11, Parent of patient aged under 8

“*NT: So how many scans would you say you took before you started, to understand them [the MRIs]?*

*C: Many. [All laugh]”* P12, 13–15 year old patient

#### Fatalism

When asked about whether they would like to take images of their child’s MRIs home, a couple of the parents responded with an attitude of fatalism, (correctly) stating that viewing the images (or not) would not change the outcomes of treatment or the way that the illness progressed. While it may ostensibly seem to be expressing powerlessness, it appeared more to be recognising the outcomes in their child were not entirely under their control, and so relieving themselves of that burden and any consequent guilt.

“*Ummmm, at the end of the day I can’t … I can’t change it. [NT: Mmmm.] There’s, there’s absolutely, it’s, nuffinkk I can do, it’s one of those things. Yaknow, lii … ittt sounds silly but it’s kindof to a certain degree, irrelevant ‘cos’ there’s nothing I can do about it, so. Ya know, it’s in the hands of the gods now. [NT: Mmmm.] So, whatever, um, whatever course they decide to take … [NT: Mmmmm.] is what [they]‘ll go down, yaknow. It [the tumour] may stay the same, or it may grow.* [ … .] *S’not really gonna change anythin’ [if I view the MRIs or not].”* P10, Parent of patient aged under 8

“*If I see the scans or I didn’t see the scans … , it doesn’t take away the fact that [they need] treatment. It won’t take, it won’t take away the fact of anyfink. It’s not gonna change anyfink, or the outcome of anyfink.*” P1, Parent of patient aged under 8

#### Humour

Humour was used by all but one patient, and all but one parent, sometimes as a way to diffuse the situation after sharing a painful, fearful or ‘serious’ memory; often when discussing the MRIs. Some parents used humour to justify why they would prefer not to bring home copies of their child’s MRIs, if given the choice.

“*Well, I’m not gonna stick it in a frame! I’m just gonna go like ‘Awww! I’ve got a picture of [Child’s] brain tumour. Everyone, who wants to look at [their] brain tumour?’ [NT laughs]*” P10, Parent of patient aged under 8

“*NT: So um, [Child], how do you think you would have felt if you weren’t allowed to see your images at all?*

*C: Don’t know really. Hard one to answer, ‘cos’ I don’t really care. [P: If the doctor had said you couldn’t see, those pictures if you wanted to...[ … ] I’m sure you would have said ‘But that’s my, picture. It’s a picture of me.’] Not a picture of me. Not -. I’d be very worried if a person looked like that. [Holds up prompt] [NT laughs]*” P7, 13–15 year old patient

“*NT (to C): What does this [the prompt] tell you? (Silence) [NT laughs]*

*C: Errrrrrr. (Pause) Um. (Pause) Don’t. Say anything, so I don’t know. It don’t talk!*

NT: [Laughs] Yeah.” P6, 8 – 12 year old patient

“*[The doctor] tries [NT laughs at parent’s expression] to explain them to us as best [they] can, but [they’re] not an expert in scans either, so...! [NT laughs, P laughs slightly]*” P8, Parent of 8 – 12 year old patient

### Seeking support

#### Social support from family and friends

Although the questions were around the individual experience of seeing images, some parents referred to support provided by family, and sometimes friends - sharing childcare, sharing their medical knowledge, and / or being emotionally supportive. Typically this was mentioned by parents of children under eight. One parent specifically discussed being able to use their child’s MRIs to increase understanding in others, so that they had more social support.

“*[Child]‘s is er, it’s a [brain tumour] … [NT: Mmmm.] and they [family and friends] say ‘What’s [that]?’ And I say [where it is]. And they go [‘What?’] [ … ] And because I’ve actually been able to showww, my close friends, the picturess [MRIs] [ … ] they go ‘Now I understand. [NT: Mmmm.] Now I understand why … , if [they get] hit in [their] face, ya, ya gotta be careful. [ … ] I’ve then made, that other person, comfortable, sooo, I can rely on, and get support from-, because it is so hard to try and just do it on your own. [Sighs]*” P3, Parent of 8 – 12 year old patient

#### Social support from other families with brain tumours

Only parents referred to support provided by other families with a similar diagnosis. These families were found helpful, as they were able to share experience and provide answers that even experienced oncology doctors, oncology nurses and palliative care nurses could not. One of the parents who mentioned being in an online support group, circulated their child’s MRIs for emotional support from other parents.

“*Yeah. [I send the images to my online support group] ‘This is what’s happened. Can you … ?’ Yeah. Yeah. Or erm ‘This is good. Look at this scan! Looks great.’ [NT laughs]*” P12, Parent of 13 – 15 year old patient

#### Differences and similarities between patients’ and parents’ coping strategies

When comparing coping strategies, there are similarities and differences in those referred to by patients and those parents (see Table 2).

**Table 2:** Which coping strategies used by patients and / or parents

Coping strategy	Used by patients	Used by parents
*Normalising*		
Maintaining normal	Y	Y
Adapting normal	Y	Y
*Maintaining hope and a sense of future*		
Maintaining hope	Y	Y
Focusing on the positive		Y
*Dealing with an uncertain future*		
‘Expertise’	Y	Y
Fatalism		Y
Humour	Y	Y
*Seeking support*		
Social support from family and friends		Y
Social support from other families with brain tumours		Y

Normalising strategies were mentioned by just over half of the families. Around half of the parents made comments suggesting they were ‘maintaining normal’, as did half of the patients; while few seemed to express comments suggesting ‘adapting normal’. These strategies tended to correspond between parents and patients from the same family. ‘Maintaining hope’, especially through using MRIs, was described by half the patients and some of the parents. Most of the patients talked about seeing the MRIs as encouraging hope or reminding them of times when the future looked worse; as did half of the parents. Only parents mentioned focusing on the positive.

In terms of ‘Dealing with an uncertain future’, all parents were ‘experts’ in their child’s condition, history, symptoms and responses; or showed clear familiarity with medical terminology, the hospital environment, processes and procedures. Only some patients made comments suggesting this. ‘Humour’ was used by most patients during the interview and all but one parent after discussing difficult times of the condition or describing the plethora of treatments and scans that they or their child had gone through.

In terms of ‘seeking support’, only parents referred to support from family and friends, or from other families with brain tumours. Though support from family and friends was clearly not possible in those that were keeping the illness, or its severity, private.

## Discussion

Past research has found many of the coping strategies identified in this article, used by patients with chronic illnesses [22–25]; children and adolescents with cancer [2, 6, 8, 26–30]; and with brain tumours specifically (low and high grade gliomas, pineoblastomas, germinoma, medulloblastoma and ependymoma) [31]. However, the authors know of no articles that have explored the role of MRIs in coping.

MRIs were reported by patient families as helping them maintain hope for the future, and focus on the positive, by showing them a time when the situation looked far worse for themselves or their child. Alternatively, some participants choose not to obtain copies of their / their child’s MRIs, in order to ‘maintain normal’, or “push back” against the intrusion of the disease on their and their family’s life [32] (p.469). Most of the patients expressing ‘maintaining normal’ were younger (8–12), whilst only older patients (13–15) expressed ‘adapting normal’ statements, which may reflect length of time since diagnosis, or maturity with age, or both.

Polar opposite ‘normalising’ strategies were possible in the same participant: finding regular visits to the hospital ‘normal’, while keeping the disease private, for example. Although this was only the case for one. However, it is not unknown that coping strategies aimed at maintaining normal often contradict each other [33]. Whether maintaining or adapting normal, using MRIs to maintain hope, or remind families that their child is in a better position, or more stable place than previously, may be useful for counselling, or professional coaching for patients, such as that offered by Macmillan Cancer Support [34].

In many cases, participants from the same family referred to the same coping strategies, although the context of the interview may have prompted a stronger correspondence than was the case. As highlighted in the first extract for ‘Adapting normal’, often a patient would give a brief addition to, or show of support for, a parent’s comments. Although past research with children with brain tumours and their parents has found that parents’ views are often in line with their children around wishing their lives would go “back to normal” or trying to create a “new normal” [31].

While no patients described social support from family, friends or other families with brain tumours, this does not necessarily mean that they do not need, have, or value it. It may have been viewed as ‘going without saying’ or perceived as beyond the scope of the research (the value to patient families of seeing MRIs).

### Strengths and limitations

As the data was not gathered to specifically explore the coping strategies used by young patients with brain tumours, and their parents, these findings provide only initial understandings of these [35]. It is possible (and quite likely) that CYP and parents engaged in more coping strategies, though did not discuss these as it was neither the focus of the research nor the interview questions. Different responses may have been given if parents and patients were interviewed independently [36], although questions were addressed to the patient first, so as to avoid them giving a perceived ‘correct response’, or deferring to their parent’s views [37]. However, having a parent present was thought to make the patients more comfortable, relaxed and more likely to provide answers [36, 37], as well as to support the development of rapport [36], and support communication between participant and researcher [36, 37].

Literature on experiences of both CYP with chronic illness and parents is scarce [38], and seems especially so for CYP with brain tumours [31], and it is hoped that this article will help address this.

Recruitment for a rare condition is often challenging [39], and the number of eligible and suitable patient families to draw on was small. However, maximum variation sampling was used to maximise diversity in participants and experiences as much as was possible, given these constraints [16]. The sample had only patients aged from 8 to 15, though experiences and understanding of a chronic illness can vary widely, according to maturity [40], further adding to diversity. A larger and more diverse cohort specifically selected to explore coping mechanisms across the desired patient population would be a useful follow-on study.

The study specifically excluded families who had recently received ‘bad news’ and it should be noted that patients with tumours for which there is no realistic chance of long term survival such as diffuse midline gliomas are not represented. Coping can change during the patient’s journey from diagnosis, through treatment, during remission and at relapse [41], and so these families would be likely to use additional or different coping strategies. Since the study did not specify the point at which their views should refer to, patients and families made comments which related to both recent and previous MRIs. It is thus not possible in our study to link the coping to specific parts of the patient journey. A study designed to specifically investigate this relationship would be valuable.

While researching the literature, the first author found it difficult to find one repository for all coping strategies, which would allow identification of all known coping strategies used by patients with brain tumours (paediatric; adolescent; or adult); those used by paediatric patients with other (similar) cancers; and those most commonly used by paediatric patients with chronic illnesses in general. Future research to create such a catalogue, or interactive tool, would appear to be warranted.

## Conclusions

Coping and finding ways to cope, are clearly used by patients and their families and are something that they wish to discuss, as they will mention these topics in conversations that are not necessarily about coping. Therefore clinicians should always allow time and space (in consultations, appointments, and in impromptu conversations on the ward) for patient families to discuss ways of coping.

MRIs were found to be used in various ways: to maintain or adapt normal; maintain hope and a sense of the future; deal with an uncertain future; and to seek support from others. Therefore clinicians should highlight the potential for MRIs to aid coping, suggesting taking copies home, if appropriate. Professional coaches or counsellors may find MRIs beneficial as a way to maintain or rebuild hope for the future by reminding families that their child is in a more stable or ‘better’ place than previously.

## Supplementary Information

**Additional file 1.**

## Data Availability

The fieldnotes and transcripts analysed during the current study are available from the corresponding author on reasonable request.

## Abbreviations

CYP: Children and Young People
MRI: Magnetic Resonance Imaging
MRIs: Magnetic Resonance Images
NF1: Neurofibromatosis Type 1
RAG: Research Advisory Group
