# Supplementary material for: The role of Magnetic Resonance Images (MRIs) in coping for patients with brain tumours and their parents: a qualitative study
Source: BMC Cancer. 2021 Sep 10;21:1013. doi: 10.1186/s12885-021-08673-z (PMC8431927; doi:10.1186/s12885-021-08673-z)
Supplement: Supplementary file 1 — Additional file 1. [file 12885_2021_8673_MOESM1_ESM.docx]

The Role of Magnetic Resonance Images (MRIs) in Coping for patients with brain tumours and their parents: A qualitative study

Natalie Tyldesley-Marshall, Sheila Greenfield, Susan J. Neilson, Martin English, Jenny Adamski, and Andrew Peet

*Interview schedule*

*(Same questions will be asked to parents, though phrased slightly differently. Prompts are within brackets)*

1

Can you tell me a bit about yourself and why you go to hospital?

2

Is it okay if I show you an example of an MRI scan of a brain tumour. [Show generic image of brain with similar tumour] Do you remember how you felt when you saw your scan for the first time? How did you feel?

(Why is that? Do you still feel this?)

[If patient does not respond, drawn picture can be given for patient to complete the doctor’s words and patient’s thoughts, or blank drawing paper and pens for younger patients]

3

What do you think your scan tells you?

(What parts are you looking at? What do they tell you? Tumour – darker/lighter/shiny area? Where the tumour is? Better means…smaller? How would you feel if you were not allowed to see this? Why is that?)

4

What do you think your scan doesn’t tell you?

(How certain / stable the changes are from last scan? Bones? Small changes or getting better? How bad the cancer is? Does the type of scanner make a difference?)

5

Do you find seeing the picture useful?

(How so? Does it help you to understand your condition better?)

6

If a new scan gave more information that may be helpful, would you want this?

(Why is that?)

7

[If yes] Would you change your mind if the information could occasionally be misleading?

8

How do you find the wait between having the scan and hearing the results?

(Far too long? Fast enough? Causing anxiety? Does it not bother you? Would you prefer to have a preliminary report?)

[If have time...

9

If you could have a copy of the scan image to take away each time, would you want that?

([If no] Would you mind telling us why that is?

[If yes] Would you mind telling us why that is? (Would you mind telling us what would you do with it?)]

10

Is there anything else you'd like to say about this?

11

Do you have any questions?
